# Morphology and Morphometry of the Acromion Process in Dried Adult Vietnamese Human Scapulae

**DOI:** 10.7759/cureus.105468

**Published:** 2026-03-18

**Authors:** Toan Nguyen Tan, Vu Nguyen Hoang, Toai Tran Cong

**Affiliations:** 1 Department of Anatomy, School of Medicine, University of Medicine and Pharmacy at Ho Chi Minh City (UMP), Ho Chi Minh City, VNM; 2 Department of Upper Limbs, Hospital for Traumatology and Orthopedics, Ho Chi Minh City, VNM; 3 Department of Anatomy, University of Medicine and Pharmacy at Ho Chi Minh City (UMP), Ho Chi Minh City, VNM; 4 Biomedical Research Center, Pham Ngoc Thach University of Medicine, Ho Chi Minh City, VNM

**Keywords:** acromion process, acromioplasty, impingement syndrome, morphometry, scapula, shoulder pain, sis, vietnamese population

## Abstract

Introduction

The shape and thickness of the acromion process are associated with the shoulder impingement syndrome (SIS), which is characterized by chronic pain and limited shoulder joint movement range. Specific morphological features of the acromion, including Bigliani type (hooked shape), increased thickness, and a reduced acromion-glenoid distance, are clinically associated with an increased risk of SIS. Thus, orthopaedic doctors and researchers need to understand the morphometry of the acromion process.

Aims and objectives

This study aims to investigate the morphology (shape and type classifications) and morphometry (length, breadth, thickness, and acromioglenoid distances) of the acromion process in dried adult Vietnamese human scapulae.

Materials and methods

This was a cross-sectional study. The study analyzed the morphology of the acromion process. The study measured the maximum length, breadth, thickness, acromio-coracoid distance, and acromio-glenoid distance.

Results

In the study, the most common shape of the acromion was the tubular shape. The thickness of the acromion process on the left and right sides was found not to be of significance (p > 0.05). The maximum length of the left and right acromion processes had an average value of 42.40 ± 6.50 mm and 43.62 ± 6.83 mm, respectively. In acromion breadth, the left side was 26.04 ± 6.44 mm, and 26.15 ± 6.75 was the average value of the right side. The acromio-coracoid distance was 37.77 ± 6.33 mm on the left and 35.60 ± 7.26 mm on the right side. The left and right acromio-glenoid distance was measured at 41.58 ± 7.12 mm and 40.43 ± 6.69 mm, respectively, with no statistically significant differences between the left and right sides for any parameters (p > 0.05)

Conclusions

The morphometric data provided - specifically, the dominance of the tubular shape and the mean acromial thickness of 6 mm - serve as a baseline for Vietnamese patients to prevent over-resection during acromioplasty, which can compromise the deltoid origin. Furthermore, the acromio-coracoid and acromio-glenoid distances provide surgeons with precise landmarks to identify pathological narrowing (<15 mm) of the subacromial space, facilitating more accurate decompression.

## Introduction

The shoulder joint is one of the most mobile yet least stable joints in the human body, relying on the harmonious interaction of bones, muscles, tendons, and ligaments to maintain functional stability and range of motion. Subacromial impingement syndrome (SIS) is frequently driven by specific anatomical variations of the acromion. The morphology of the acromion is a critical factor in the pathogenesis of shoulder impingement syndrome (SIS). Specifically, a Type III (hooked) acromion according to the Bigliani classification, increased acromial thickness, and reduced acromion-glenoid distances have been shown to significantly narrow the subacromial space, leading to mechanical compression of the supraspinatus tendon [1, 2]. While the Bigliani classification remains the clinical standard, recent metrics such as the critical shoulder angle (CSA) offer higher predictive value for pathology. Furthermore, scapular morphology exhibits significant ethnic variation; for instance, South Asian populations demonstrate a higher prevalence of Type II curved acromia (approx. 54%), whereas Type III hooked variations are more frequently documented in symptomatic Caucasian cohorts, suggesting that population-specific anatomical baselines are essential for accurate diagnosis [3, 4]. Among its various etiologies, SIS is one of the most frequent causes of chronic shoulder pain and functional limitation [5, 6].

The morphology of the acromion process plays a crucial role in the pathophysiology of SIS. Variations in acromial shape and dimensions can alter the subacromial space, predisposing to rotator cuff compression and subsequent tendon degeneration [6-8]. Previous studies have proposed several classifications of the acromion process [9]. These variations may differ across ethnicities due to genetic and environmental factors, including occupational or lifestyle differences. Recently, the development of imaging techniques, such as magnetic resonance imaging (MRI) and computed tomography scan (CT scan) [6, 8, 10], has provided better tools for researchers to analyze the anatomical structure and features of the acromion process more accurately. Despite numerous morphometric investigations in different populations, data on Vietnamese adults remain limited. Establishing reference values for the acromial morphology in this population is essential for both anatomical education and surgical planning, particularly for procedures such as acromioplasty. Accurate population-specific data is critical to prevent the over-diagnosis of impingement or the over-resection of bone, which can lead to deltoid origin failure in different ethnic cohorts. Therefore, this study aimed to analyze the morphology and morphometric characteristics of the acromion process in dried adult Vietnamese scapulae. While clinical imaging often reflects soft-tissue changes, the use of dried bones allows for high-precision direct measurements of cortical bone morphology without the distortion or slice-thickness limitations inherent in CT or MRI. Although the sex and exact age of the specimens were unknown - a common constraint in osteological studies - these findings provide a foundational anatomical baseline for the Vietnamese population and allow for comparison with previously published data from other global cohorts.

## Materials and methods

This descriptive cross-sectional study was conducted on 95 dried adult scapulae (48 left and 47 right) obtained from the formalin-fixed cadaveric collections of the Department of Anatomy, Faculty of Medicine, University of Medicine and Pharmacy at Ho Chi Minh City (UMP).

Dry bone specimens were selected for this study over clinical imaging modalities (such as CT or MRI) to allow for direct, high-precision osteometric measurements. This approach eliminates the 'partial volume effect' and potential projection artifacts inherent in radiographic imaging, providing a true 1:1 anatomical representation of the cortical margins.

A recognized limitation of this collection is that the specimens were of undetermined sex and age. Given that the scapula exhibits significant sexual dimorphism, this study focuses primarily on morphological patterns (shape and type) and proportional indices, which are less sensitive to sex-based size variations than absolute linear dimensions. Furthermore, the inclusion of 95 specimens provides a sufficiently robust sample size to establish a general population baseline for the Vietnamese adult population, consistent with similar osteological studies in other regions [4]. Scapulae showing fractures, visible deformities, marked degenerative changes, or juvenile features - such as incomplete epiphyseal fusion - were excluded. Only well-preserved adult scapulae with intact acromial processes were included.

**Figure 1:**
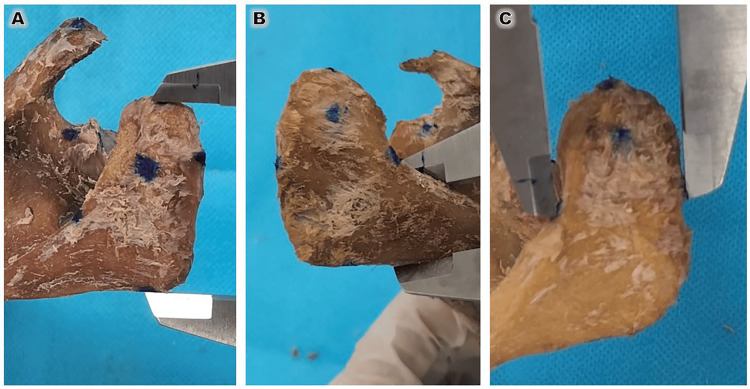
Morphological classification of the acromion process (superior view). (A) Quadrangular, (B) triangular, and (C) tubular shapes, classified according to the criteria established by Getz et al. (1996) [11]. The images were taken in the Department of Anatomy, Faculty of Medicine, University of Medicine and Pharmacy at Ho Chi Minh City (UMP).

The acromion process of each scapula was examined macroscopically from two distinct anatomical planes. First, the acromial shape was classified from a superior (top-down) view into three categories - quadrangular, triangular, and tubular - following the morphological criteria established by Getz (1996) (Figure 1) [11]. Second, the acromial type was evaluated from a lateral perspective based on the degree of inferior curvature (Type I: flat; Type II: curved; Type III: hooked). While this classification was originally proposed by Bigliani (1986) [12] for outlet radiographs, it has been widely validated for use in osteological specimens by Natsis (2007) [13], who demonstrated that direct visual inspection of dry bone provides a more precise assessment of the subacromial enthesis. Each specimen was independently evaluated by two trained anatomists to ensure the objectivity of the qualitative classifications. To standardize the identification of acromial types (Type I: flat, Type II: curved, and Type III: hooked), the observers utilized a reference set of standardized anatomical models based on the Natsis [13] adaptation of the Bigliani criteria.

To document morphological variations and ensure reproducible qualitative analysis, each specimen was photographed using a Nikon D5500 digital camera equipped with a 50mm fixed focal length lens (Nikon Corporation, Tokyo, Japan) to minimize edge distortion. The camera was secured to a professional-grade tripod and aligned using a dual-axis spirit level to ensure the optical axis was strictly perpendicular to the plane of the acromion, thereby eliminating parallax error.

Specimens were positioned at a standardized distance of 30 cm on a matte black background to enhance border contrast. A medical-grade metric scale was placed in the same focal plane as the acromial process for digital calibration. To maintain consistency across the dataset, lighting was standardized using a dual-source LED arrangement to eliminate harsh shadows on the subacromial surface. All measurements were recorded to the nearest 0.01 mm, and the mean of three consecutive readings was used for final statistical analysis.

**Figure 2:**
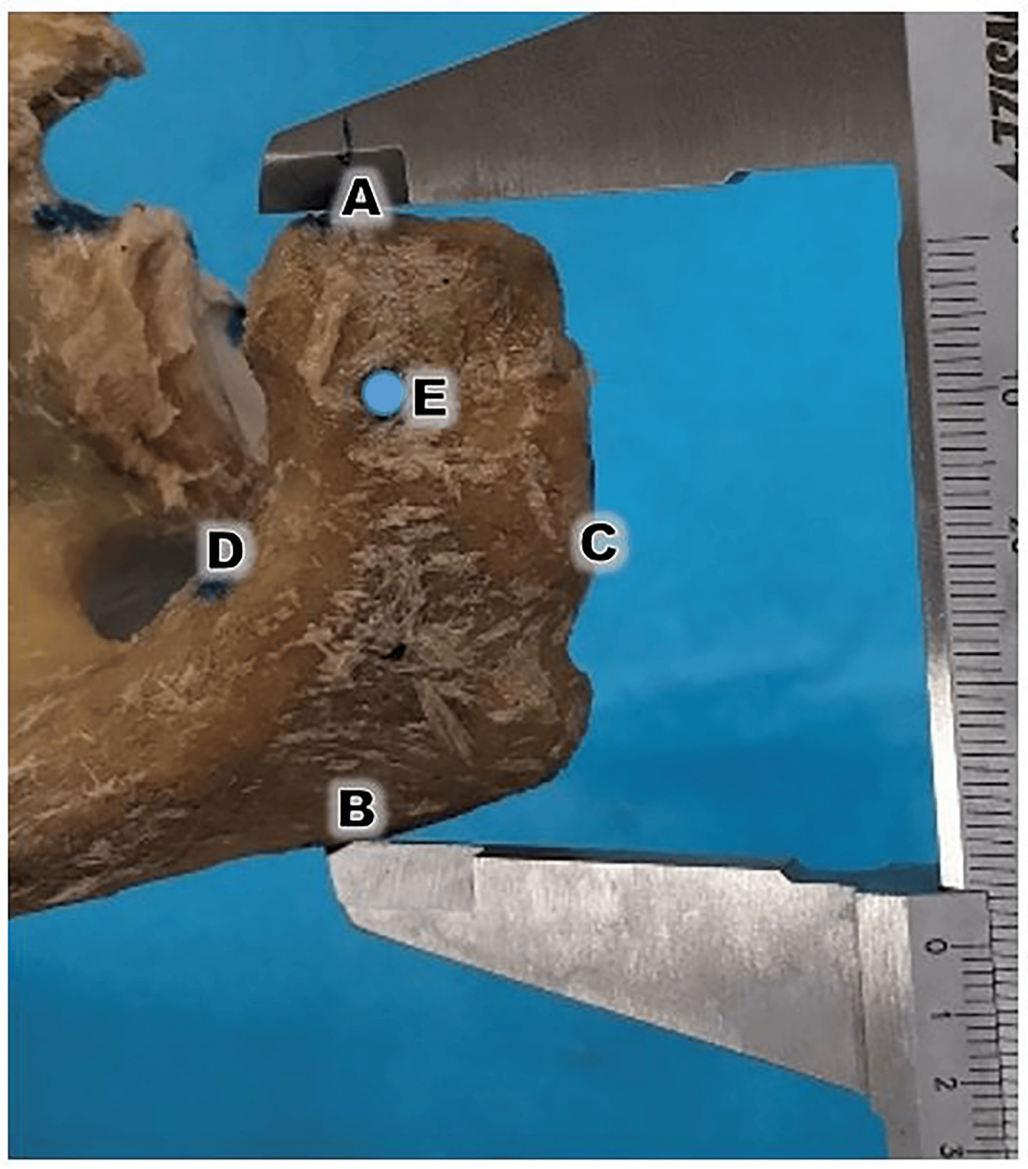
Morphometric measurements Morphometric measurements were standardized according to the landmarks established by Singroha et al. [16]. Maximum Acromial Length (AB): This was measured from the most anterior point of the acromion (Point A) to the posterior-lateral angle of the acromion (Point B). Maximum Acromial Breadth (CD): This represents the greatest distance between the medial and lateral borders of the acromion, measured perpendicular to the length (AB). Acromial Thickness (E): This was measured at a standardized point 1 cm posterior to the anterior border and 1 cm medial to the lateral border.

The following morphometric parameters were recorded [14, 15]. Morphometric measurements were performed using standardized landmarks to ensure comparability with global anatomical data. Maximum acromial length (Figure 2, length AB) was measured from the most anterior point of the acromion to the posterior-lateral angle (the 'acromial angle'). Maximum acromial breadth (Figure 2, length CD) was defined as the greatest distance between the medial and lateral borders, measured perpendicular to the length (AB) at the midpoint of the acromial process. These definitions follow the protocols established by Singroha [16] for dry scapulae. Acromial thickness (Figure 2, Point E) was measured 1 cm posterior to the anterior border of the acromion. This specific site was selected because the anterior third of the acromion is the primary area involved in subacromial impingement and is the critical region targeted during surgical acromioplasty.

**Figure 3:**
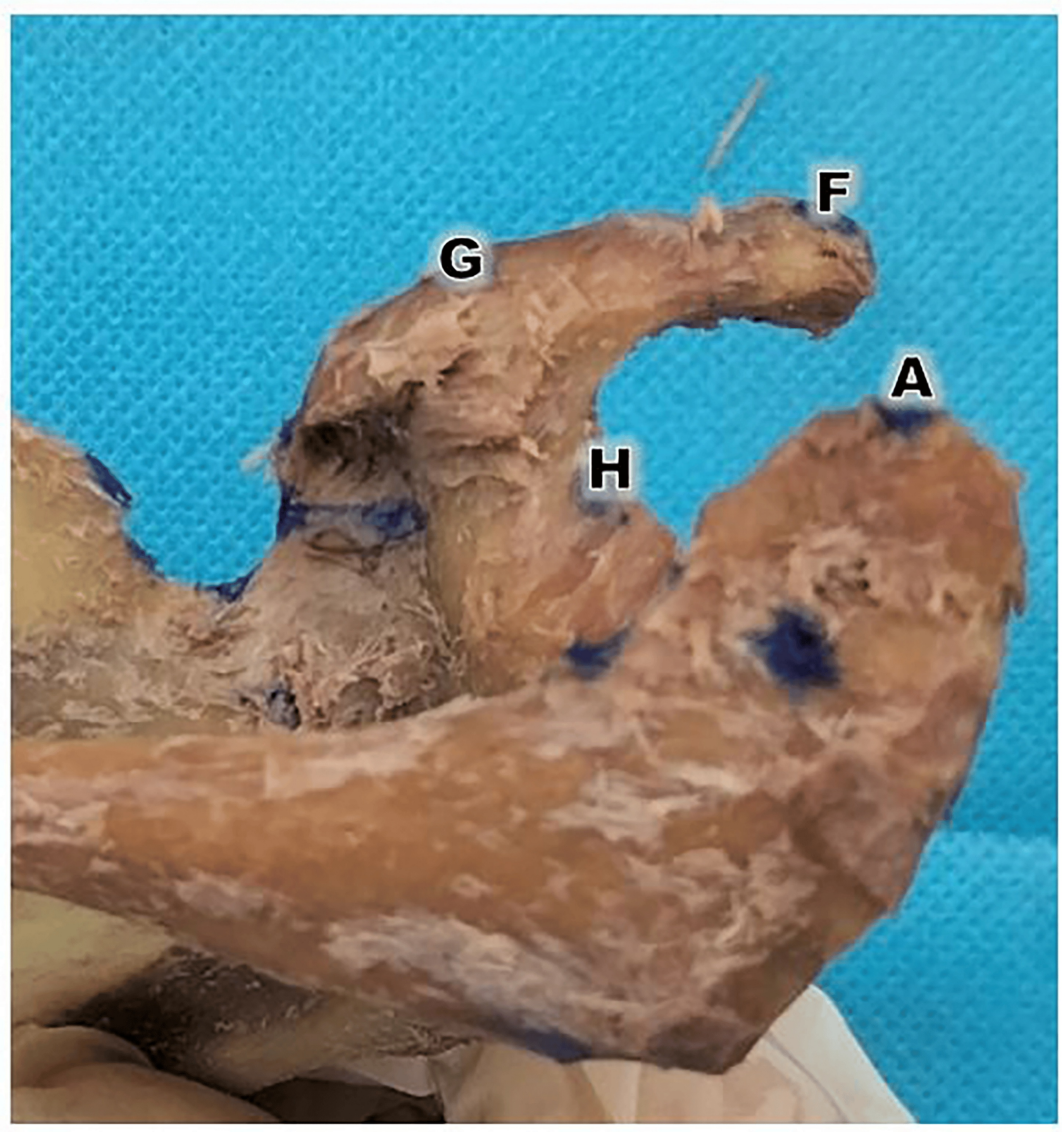
Acromion process distance with relevant structure The acromio-coracoid distance (AF length) was measured from the tips of the acromion process (Point A) to the coracoid process (Point F). The acromio-glenoid distance (AG legth) was measured from the tip of the acromion process (Point A) to the supraglenoid tubercle (Point G). H: the supraglenoid tubercle apex, which is the highest point on the supraglenoid tubercle, often serving as the origin point for the long head of the biceps brachii tendon

To ensure high reproducibility, the acromio-coracoid (Figure 3, length AF) and acromio-glenoid (Figure 3, length AG) distances were measured between clearly defined osteological landmarks. The acromio-coracoid distance (AF) was defined as the shortest linear distance between the most anterior point of the acromion process (Figure 3, Point A) and the most superior-lateral aspect of the apex of the coracoid process (Figure 3, Point F). The acromio-glenoid distance (Figure 3, length AG) was defined as the linear distance from the most anterior point of the acromion (Figure 3, Point A) to the center of the supraglenoid tubercle (Figure 3, Point G), which serves as the attachment site for the long head of the biceps brachii. These landmarks were identified through manual palpation of the dry bone and verified by two independent observers. By using the apex of the coracoid and the center of the supraglenoid tubercle, we minimized the measurement variability associated with the broad surfaces of these processes [15].

Statistical analysis

Continuous variables were expressed as mean ± standard deviation (SD) and compared between left and right sides using the Mann-Whitney U test, as the morphometric data did not meet normal distribution criteria. Categorical variables were analyzed using the Chi-square test. Statistical significance was defined as p < 0.05, and exact p-values are reported for all comparisons. All analyses were performed using GraphPad Prism version 8.0 (GraphPad Software, Boston, USA).

Ethical statement

The study protocol was approved by the Biomedical Research Ethics Committee of the University of Medicine and Pharmacy at Ho Chi Minh City (UMP) (IRB-VN01002/IORG0008603/FWA00023448; Decision No. 247/HĐĐĐ-ĐHYD, dated January 29, 2024). All cadaver donors had provided written informed consent for the use of their bodies in anatomical education and scientific research. The study was conducted in accordance with the Declaration of Helsinki.

## Results

**Figure 4:**
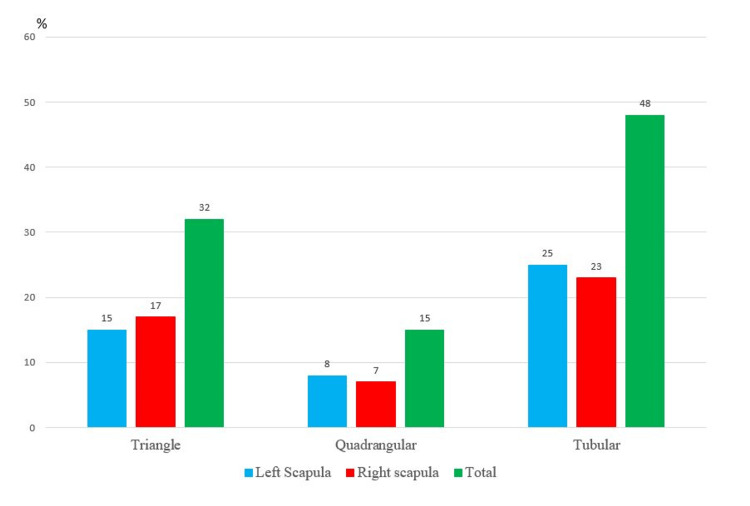
Prevalence of the different acromion shapes in the study This bar chart illustrates the prevalence of the three different anatomical shapes of the acromion process - quadrangular, triangular, and tubular - as observed in the study population. The data is presented separately for the left scapula (blue bars) and the right scapula (red bars) to compare the incidence between the two sides. The total count (green bars) for each shape is also shown. The figure clearly demonstrates that the tubular shape was the most common overall, while the triangular shape was the least common. The number indicates the frequency for each category.

In this study, we found that the most frequent shape of the acromion process was the tubular shape on both left and right sides. The second most common shape was quadrangular on the right side, while the triangular shape was the least common on the left side as well as the right side (as in Figure 4, Table 1). The significance of the incidences of various shapes of the acromion process was found not significant (Chi-square test p = 0.99).

**Table 1:** Summary of the different shapes of acromion process (n (%)) This table summarizes the distribution and percentage frequency of the three distinct anatomical shapes of the acromion process (quadrangular, triangular, and tubular) as observed across 95 human dry scapulae. The data is categorized by the side of the body: left scapula and right scapula. The results indicate a non-significant difference in shape distribution between the two sides, as shown by the chi-square test p = 0.99.

Shape	Left Scapula	Right Scapula	Total	Chi-square test p = 0.99
Quadrangular	15 (15.79%)	17 (17.89%)	32 (33.68%)	
Triangular	8 (8.42%)	7 (7.37%)	15 (15.79%)	
Tubular	25 (26.32%)	23 (24.21%)	48 (50.53%)	
Total	48 (50.53%)	47 (49.47%)	95 (100%)	

**Figure 5:**
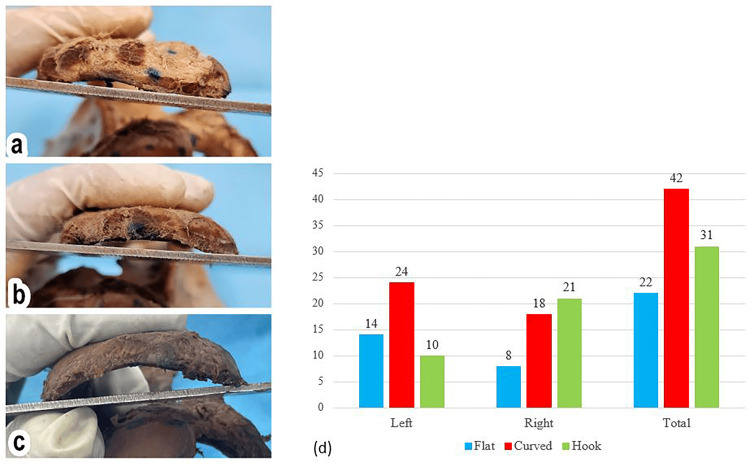
Prevalence of acromion types by side The figure presents a: flat type (I), b: curved type (II), and c: hook type (III) of the acromion process. d: bar chart illustrating the percentage distribution of acromial types (Type I: Flat, Type II: Curved, and Type III: Hooked). While minor variations between the sides are visible, Chi-square analysis confirms no statistically significant difference in type distribution between the left and right scapulae (p = 0.95). Data labels include absolute counts (n) for independent evaluation.

**Figure 6:**
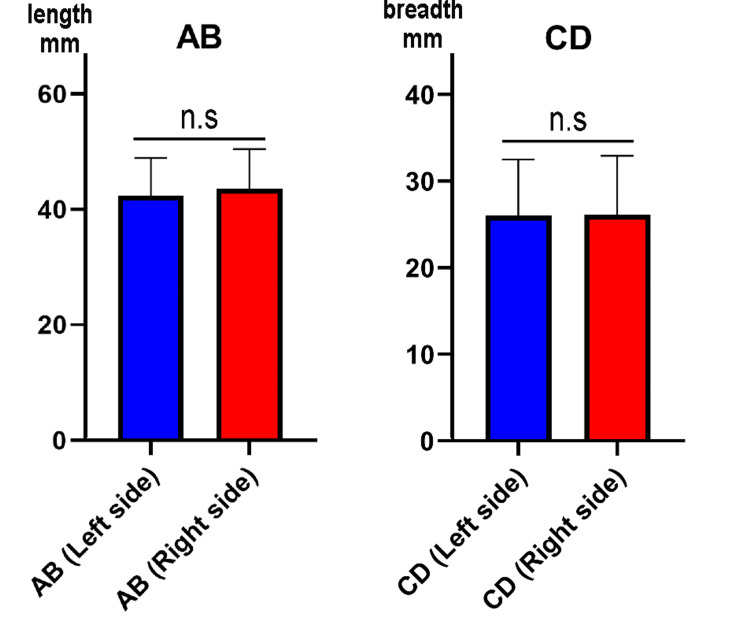
Comparision of acromion process length and breadth The figure consists of two paired bar graphs comparing the mean ± standard deviation of the two key morphometric parameters of the acromion process - length (Figure 2, AB length) and breadth (Figure 2, CD length) - between the left and right scapulae. Left panel (AB): Compares the mean acromion process length (AB length) for the left side (blue) and the right side (red). Right panel (CD): Compares the mean breadth of the acromion process (CD length) for the left side (blue) and the right side (red). The label "n.s" (no significance) above each pair of bars indicates that the statistical analysis found no statistically significant difference (Mann–Whitney U test p > 0.05) between the mean measurements of the left and right sides for both the acromion process length (AB) and breadth (CD). Y axis mm = millimeter

**Figure 7:**
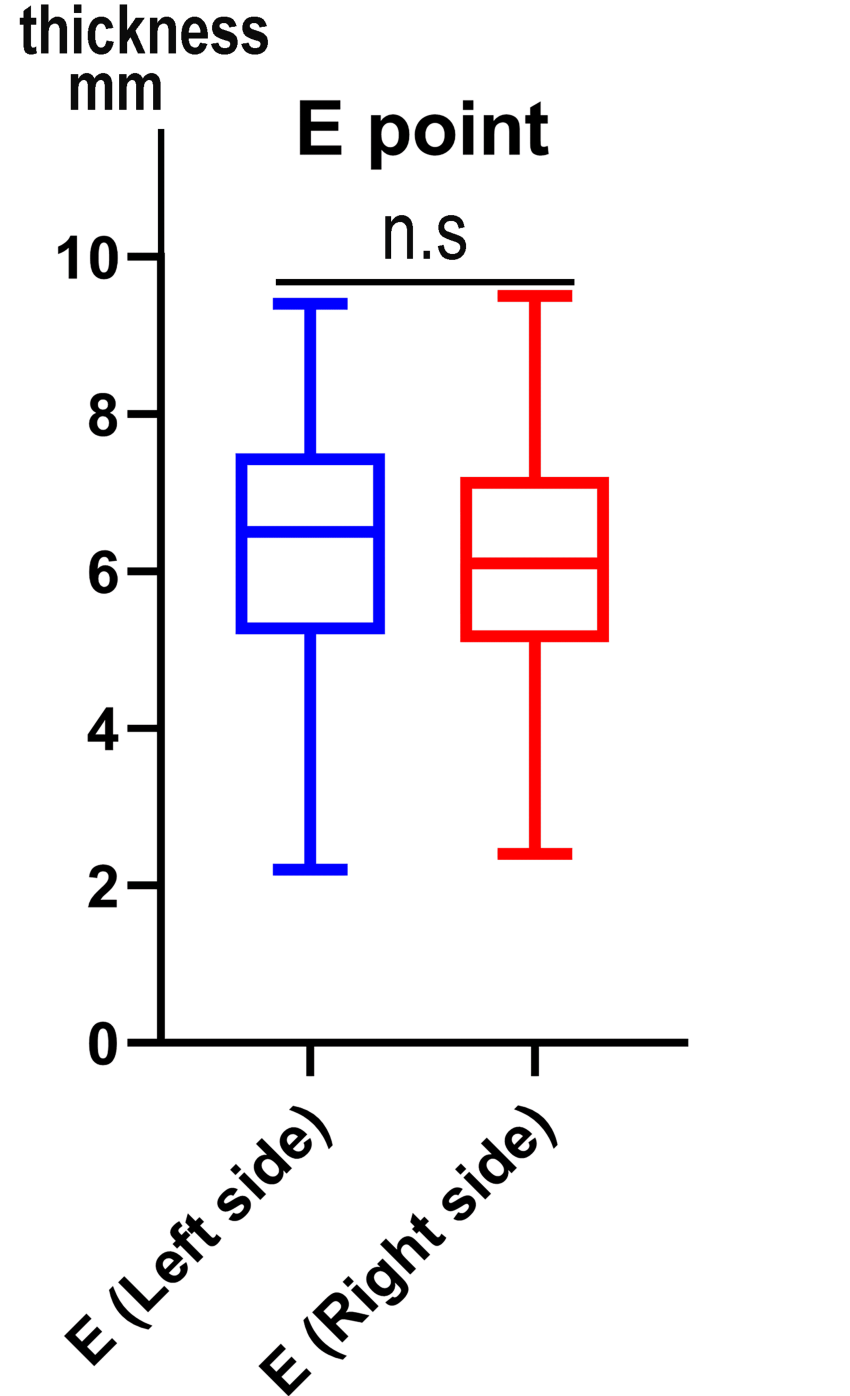
Comparision of acromion thickness at 1 cm behind the tip of acromion This box-and-whisker plot illustrates the distribution of thickness for the left and right sides. Individual data points plotted beyond the whiskers represent outliers, reflecting the wide morphometric range (2.18-9.49 mm) observed in this cohort. No statistically significant (n.s) difference was found between sides (Mann–Whitney U test, p > 0.05). Y axis mm: millimeter.

This study found that the curved type was prominent in the left scapula bone, while in the right scapular bone, the “hook” type was more than the other types (as shown in Figure 5). The study found that the average length of the left acromion process (AB length) was 42.40 ± 6.50 mm, with a range of 21.50 mm to 57.50 mm. The right acromion process had an average length of 43.62 ± 6.83 mm, with a range from 28.10 mm to 68.90 mm. With the breadth of the acromion process (CD length), the left side has an average of 26.04 ± 6.44 mm (from 17.09 mm to 53.01 mm), while the right side has an average value of 26.15 ± 6.75 mm (range from 14.42 mm to 52.01 mm) (Figure 6). The present study also found that the thickness of the left acromion process measured between 2.18 mm and 9.43 mm, with an average of 6.33 ± 1.79 mm. On the other hand, the right side had an average value of 6.08 ± 1.57 mm, ranging from 2.42 mm to 9.49 mm. The data showed that the thickness between the left and right sides of the acromion process was not significant (Figure 7). Table 2 presents the parameters of the acromion process.

**Table 2:** Parameters of the acromion process This table presents a comparative analysis of five key morphometric parameters of the acromion process. The parameters measured include: acromion process length (Figure 2, AB length): the distance from the tip of the acromion process to the midpoint of its posterior border. Breadth of the acromion process (Figure 2, CD length): the distance between the lateral and medial borders of the acromion at the midpoint. Acromion thickness (Figure 2, E point): the vertical thickness measured at a point 1 cm below the acromial tip. Acromion-coracoid distance (Figure 3, AF length). Acromion-glenoid distance (Figure 3, AG length). The data is separated into left scapular and right scapular sides, showing the mean ± standard deviation and minimum-maximum range for each measurement. The p-value for each parameter indicates that there is no statistically significant difference between the measurements on the left and right sides (Mann-Whitney U test, p > 0.05 ).

Parameters	Left Scapula (n=48)	Right Scapula (n=47)	Mean Diff. (95% CI)	Effect Size (Cohen’s d)	p-value
Acromion length (AB)	42.40±6.50	43.62±6.83	−1.22 (−3.5,1.1)	0.18	0.53
Acromion breadth (CD)	26.04±6.44	26.15±6.75	−0.11 (−2.3,2.1)	0.02	0.54
Acromion thickness (E)	6.33±1.79	6.08±1.57	0.25 (−0.3,0.8)	0.15	0.5
Acromio-coracoid (AF)	37.77±6.33	35.60±7.26	2.17 (−0.2,4.5)	0.32	0.12
Acromio-glenoid (AG)	41.58±7.12	40.43±6.69	1.15 (−1.3,3.6)	0.17	0.53

**Figure 8:**
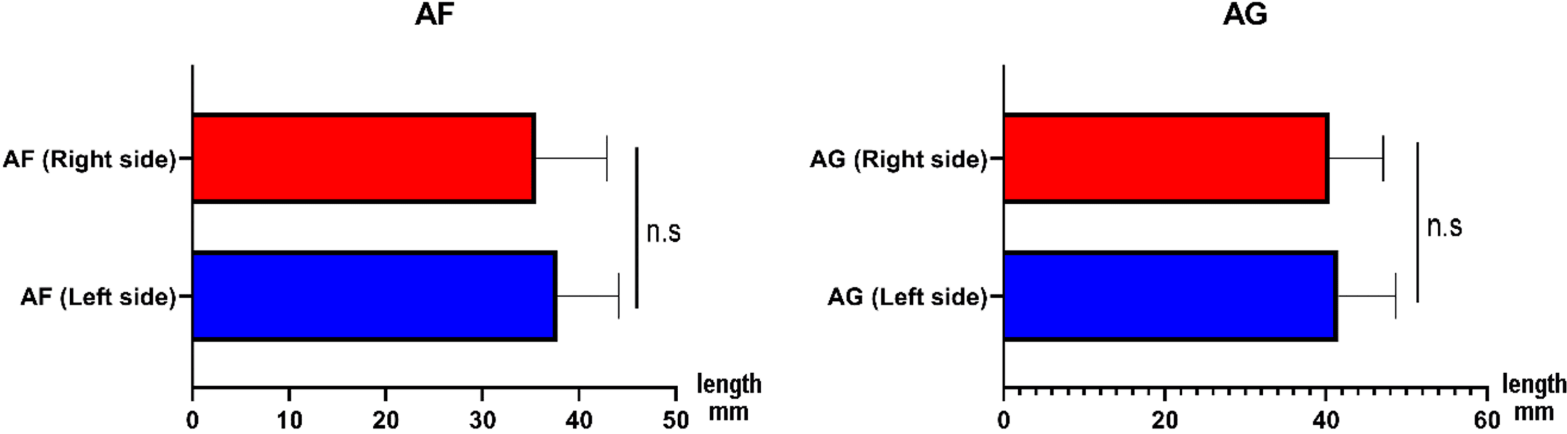
Comparision of acromion-glenoid distance (AG) and acromion-coracoid distance (AF) This figure consists of two horizontal bar charts comparing the measured distances (in mm) between anatomical points on the left and right sides of the shoulder. AF panel: This chart compares the distance between the acromion and the coracoid process (Figure 3, AF length) on the right side (red) and the left side (blue). The bar represents the mean distance, and the error bars indicate the standard deviation (or similar measure of variability). The annotation "n.s" (no significance, Mann–Whitney U test, p > 0.05) indicates that there is no statistically significant difference in the AF distance between the right and left sides. AG Panel: This chart compares the distance between the acromion and the glenoid cavity (Figure 3, AG length) on the right side (red) and the left side (blue). The bar represents the mean distance, and the error bars indicate the standard deviation (or similar measure of variability). The annotation "n.s" (no significance, Mann–Whitney U test, p > 0.05) indicates that there is no statistically significant difference in the AG distance between the right and left sides.

We then measured the distance between the acromion process and the coracoid bone on both left and right scapular bones. The mean AF distance on the left side was 37.77 ± 6.33 mm, with a range from 23.99 mm to 54.10 mm, while the mean AF distance of the right side was 35.60 ± 7.26 mm. The statistical result showed that the differences between the AF distances on the left and right sides were not significant. The average acromio-glenoid distance (AG) of the left side and right side was 41.58 ± 7.12 mm and 40.43 ± 6.69 mm, respectively. And the result showed that the difference was not significant, with p = 0.53 (Figure 8).

## Discussion

Many studies about the morphology of the acromion process have been done. However, the association between acromion morphology and shoulder impingement syndrome (SIS) remains complex. Then, clinicians and researchers still thought that there may be a correlation between acromial morphology, rotator cuff tears, and SIS. The present study provides the first morphometric data on the acromion process in Vietnamese adults. The tubular shape was the most prevalent, occurring in 50.53 % (n = 48/95) of the specimens, followed by quadrangular 33.68% (n=32/95) and triangular 15.79% forms (n=15/95). Our results reveal a distinct morphological profile for the Vietnamese acromion compared to other Asian populations.

In a previous study, Mansur et al. [17] had reported three different shapes of the acromion process in their study, and the most common shape was the quadrangular shape. Akhtar et al [5] also found that the quadrangular shape was one of the three most common shapes in their study. However, in the present study, the most common acromial shape was tubular (50.53%). These results contrast with previous findings from Nepalese and Indian populations, where the quadrangular type predominated. Such differences may reflect ethnic, genetic, or occupational variations influencing scapular development. The predominance of the Tubular acromion shape in our study population suggests a specific morphometric trend that may be influenced by both genetic predisposition and biomechanical loading. From a genetic perspective, acromial shape is largely determined during ossification of the meso-acromion; however, biomechanical factors cannot be overlooked. The tubular morphology may provide a different distribution of surface area for the origin of the deltoid muscle compared to flatter shapes.

In relation to population biomechanics, some studies suggest that repetitive overhead activity or mechanical stress on the acromioclavicular ligaments can trigger bone remodeling. However, the lack of a significant difference between the left and right sides (p=0.99) in our study suggests that these shapes are likely congenital or developmental rather than a result of handedness or unilateral physical activity.

We also compared the incidence of these three acromion shapes with other studies (Table 3). We hope our results serve as basic reference parameters for Vietnamese clinicians and SIS operative treatment.

**Table 3:** Comparison of acromial shape prevalence across different populations This table compares the percentage of triangular, quadrangular, and tubular acromion shapes in the current Vietnamese cohort with previously published data from Nepalese and Indian populations. Notably, the Vietnamese population demonstrates a unique predominance of the tubular shape (50.53%), contrasting with the quadrangular predominance observed in other regional groups.

Authors	Study Population	Triangular (%)	Quadrangular (%)	Tubular (%)
Mansur et al. [17]	Nepalese	36.76%	52.94%	10.29%
Sinha et al. [18]	Indian	31.14%	55.73%	13.11%
Akhtar et al. [5]	North Indian	38.34%	51.67%	9.99%
This study	Vietnamese	15.79%	33.68%	50.53%

The morphology of the acromion process is a well-established intrinsic risk factor for developing subacromial impingement syndrome (SIS), which is characterized by the mechanical compression of subacromial structures, primarily the rotator cuff tendons, against the inferior surface of the acromion. Curved or thicker acromia reduce the subacromial space, increasing friction on the supraspinatus tendon and bursa, predisposing to rotator cuff tears. Tangtrakulwanich and Kapkird [19] in their study had identified risk factors associated with SIS, such as smoking, occupation, sleeping position, and acromion shape. Apart from the lateral acromion, previous studies point out that the coraco-acromial ligament and the acromio-clavicular joint are possible sites for SIS. The acomion thickness also plays an important role in SIS and its operative treatment called acromioplasty, in which doctors cut out the anterior one-third of the process. The mean acromial thickness in this study (6.33 ± 1.79 mm on the left and 6.08 ± 1.57 mm on the right) was comparable to those reported in Indian and Nepalese series, suggesting a consistent anatomical dimension across Asian populations. The wide range of acromial thickness observed in our study (2.18-9.49 mm) warrants clinical consideration. The extreme values (outliers) likely reflect age-related remodeling rather than measurement error. The acromial thickness in this study exhibited a wide range (2.18-9.49 mm). The lower extreme (2.18 mm) likely reflects natural anatomical variation or age-related osteoporotic thinning, as all specimens were screened for structural integrity to rule out significant bone erosion or taphonomic damage. Conversely, the upper range (near 9.49 mm) may be associated with degenerative remodeling or the presence of enthesophytes at the coracoacromial ligament attachment.

From a clinical perspective, these variations are critical for acromioplasty. A thickness threshold of approximately 7-8 mm is often considered the 'safe zone' for bone resection. Our findings suggest that individuals at the lower end of the distribution (<5 mm) are at a significantly higher risk of iatrogenic acromial fracture if standard resection depths are applied. Therefore, preoperative planning using 3D-CT to assess the Point E thickness is essential to customize the surgical approach.

In our study, the curved and hook type was prominent, suggesting that it may play an important role in the causes of shoulder pain. The process thickness could become thicker by bone remodeling in the aging process or ligaments inflamation. Acromion thickness could affect the vertical dimension of the subacromial space, so that the thickness of the acromion was a consideration for treating SIS. When performing acromionplasty, doctors will remove the entire anterior section of the acromion. To further investigate the factors affected by the subacromial space, we measured the acromio-coracoid and acromio-glenoid distances. No significant side differences were observed in any measured parameters (p > 0.05), consistent with the bilateral symmetry commonly noted in skeletal morphology. The measured acromio-coracoid (AF) and acromio-glenoid (AG) distances also agreed with prior reports, supporting their use as reliable anatomical landmarks during acromioplasty and arthroscopic procedures. These parameters are relevant because excessive narrowing of the coracoacromial interval (<15 mm) has been linked to SIS symptoms. The acromion thickness, AG distance, and AF distance in the study were nearly similar to the results of Sinha et al [18] and Akhtar et al [5]. The coraco-acromial ligament, represented by the coracoacromial distance (AF distance), can be useful when doing ligament excision, which can reduce discomfort in impingement patients. On the other hand, the acromion process starts to degenerate when the distance between the coraco-acromial arch and supraglenoid tubercle is more than 15 mm [5, 20]. However, thicker or curved acromion shapes may reduce the subacromial space, increasing friction on the supraspinatus tendon and bursa during abduction. This can contribute to tendon degeneration, rotator cuff tears, and chronic shoulder pain.

From a clinical standpoint, understanding acromial morphology is critical for preoperative assessment and surgical planning. During acromioplasty, removal of the anteroinferior acromial segment must consider both acromial shape and thickness to avoid compromising shoulder biomechanics. Morphometric reference data, such as those presented here, may thus assist orthopedic surgeons in tailoring interventions to the Vietnamese population.

Strengths and limitations

A major strength of this study is the systematic osteological measurement of a relatively large number of dried scapulae, allowing for direct, precise, and reproducible morphometric evaluation. However, several limitations must be explicitly acknowledged.

Unknown Specimen Sex: The sex of the skeletal remains was not documented in the collection records. This prevents the assessment of potential sex-based dimorphism in acromial morphology, which may influence clinical interpretations.

Lack of Soft-Tissue Correlation: Because the study utilized dried bones, it was not possible to correlate the osteological measurements with soft-tissue structures (such as the coracoacromial ligament or rotator cuff tendons) or evaluate dynamic subacromial relationships during shoulder motion.

Geographic Specificity: The sample was derived from a single regional anatomical collection. While providing valuable data, this focus may limit the generalizability of the findings to the broader, more diverse Vietnamese population.

## Conclusions

In summary, this study enriches the regional anatomical database by documenting the morphology and morphometric characteristics of the acromion process in Vietnamese adults. These findings may support anatomical teaching, forensic identification, and clinical applications related to shoulder impingement and reconstructive surgery.
